# Grandparenting and Health in Later Life: Intensity and Age, Gender, and Urbanicity Variations

**DOI:** 10.1093/geroni/igaa057.084

**Published:** 2020-12-16

**Authors:** Yue Zeng, Yu-Chih Chen

**Affiliations:** The University of Hong Kong, Hong Kong, China

## Abstract

Grandparents play increasingly indispensable roles in providing family care. Although prior cross-sectional studies have shown a positive link between grandparenting and health, we know little about the optimal engagement level of grandparenting, its longitudinal implications, and variations on health outcomes. Guided by the role theory and social model of health promotion, we used propensity score analysis and multilevel analysis with three biennial waves of China Health and Retirement Longitudinal Study (2011-2015) to examine the longitudinal impacts of grandparenting intensity (no, low-, moderate-, and high-intensity) on health (mobility limitations, depressive symptoms, cognition, and self-rated health) among 4,925 older adults aged 45 and older, and how these impacts vary by age (45-59/60+), gender (male/female), and urbanicity (urban/rural). Controlling for the baseline sociodemographics (e.g., education and income), health limitations (e.g., ADLs and IADLs), and health behaviors (e.g., drinking and smoking), our results showed that, compared to no grandparenting, grandparenting provided at a moderate level was associated with fewer mobility limitations, lower depressive symptoms, and better cognition. Furthermore, grandparenting had a positive impact on physical, mental and cognitive health for 60+ older adults but not for the young-old. Both older males and females showed better physical health if they provided care at a low level, but older females showed better self-rated health. Older adults in the rural area showed better physical health; for the urban area older adults, better cognition. Findings suggest that policies aimed at supporting grandparents should consider the optimal threshold and variations by age, gender, and urbanicity.

